# Development and validation of a multi-modality system combining radiomics and deep learning for predicting mid-pregnancy complications and enabling timely pregnancy care

**DOI:** 10.3389/fped.2025.1716073

**Published:** 2025-12-18

**Authors:** Juan Guo, Yuhong Huang, Zhiwei Zhang, Baoqiang Shi, Shuxian Xi, Yuanyuan Mai, Yan Liang, Zhizhen Guo, Lantian Shang

**Affiliations:** 1 Pingliang City Maternity and Child-care Hospital, Pingliang, China; 2 Department of Breast Cancer, Cancer Center, Guangdong Provincial People’s Hospital (Guangdong Academy of Medical Sciences), Southern Medical University, Guangzhou, China; 3 SingularityFlow Co. Ltd., Beijing, China

**Keywords:** deep learning, gestational diabetes mellitus, hypertensive disorders of pregnancy, radiomics, ultrasound

## Abstract

**Introduction:**

To improve the early prediction of hypertensive disorders of pregnancy (HDP) and gestational diabetes mellitus (GDM), we developed and validated an artificial intelligence (AI) model. This initiative was driven by the insufficient accuracy of current clinical tools. Our study aimed to determine whether integrating radiomics and deep learning features from first-trimester ultrasound scans could enhance predictive performance.

**Methods:**

A total of 213 pregnant women who underwent ultrasound at 8 weeks of gestation were enrolled. Clinical data, radiomics features, and deep learning features were collected. Imaging features were selected using LASSO regression. Four predictive models were developed: a clinical model, a radiomics model, a deep learning model, and a fusion model combining all feature types. Model performance was evaluated on an independent test set using metrics including AUC, sensitivity, specificity, calibration, and decision curve analysis.

**Results:**

In the training cohort, all models demonstrated excellent discriminatory ability, with the combined model achieving the highest AUC of 0.987 (95% CI: 0.9733–0.9999), followed by the DLR model (AUC = 0.985). The clinical model (AUC = 0.941) and radiomics model (AUC = 0.939) also performed well. In the test cohort, the combined model maintained superior performance with an AUC of 0.963 (95% CI: 0.9152–1.0000), significantly outperforming all single-modality models. Overall, the combined model exhibited optimal and stable predictive performance across both training and test datasets.

**Discussion:**

This enables accurate early prediction of HDP and GDM. This non-invasive tool supports tailored prenatal care, with potential to improve outcomes. Further validation in diverse groups is needed.

## 1 Introduction

Hypertensive disorders of pregnancy (HDP) and gestational diabetes mellitus (GDM) are among the most prevalent complications affecting maternal and fetal health and are associated with significant long-term consequences, including preeclampsia and fetal growth restriction (1, 2). Notably, despite their distinct clinical presentations, HDP and GDM share a common pathophysiological foundation characterized by placental dysfunction, systemic inflammation, and endothelial dysfunction, which collectively contribute to profound insulin resistance. This overlapping biological rationale justifies their modeling as a combined outcome in predictive studies, as risk factors and early pathological processes for one condition often predispose to or exacerbate the other (3, 4). Early identification of high-risk pregnancies enables timely interventions, improving maternal and neonatal outcomes. Recent studies suggest that first-trimester metabolic, vascular, and inflammatory changes are independent predictive factors of mid-pregnancy complications (5). However, current screening methods rely on maternal history, biochemical markers, and lifestyle factors, which may lack sufficient predictive power. Due to the increasing prevalence of HDP and GDM worldwide, there is a critical need for non-invasive and early-stage predictive models that enable personalized pregnancy management.

Recent advances in medical imaging and artificial intelligence (AI) have demonstrated the potential of deep learning and radiomics in predicting disease outcomes by extracting high-dimensional, quantitative imaging biomarkers (6, 7). In prenatal care, ultrasound is widely accessible, non-invasive, and routinely used for fetal and maternal assessments (8). The integration of deep learning with first-trimester ultrasound has shown promising results in identifying subtle imaging patterns that may reflect early vascular dysfunction and metabolic abnormalities associated with pregnancy complications (9). Additionally, radiomics enables the extraction of texture, shape, and intensity features from ultrasound images, capturing microstructural variations that are imperceptible to the human eye (10, 11). By using these imaging-derived biomarkers along with maternal clinical parameters, fusion models can improve the accuracy of risk prediction for mid-pregnancy HDP and GDM (12).

Beyond early risk stratification, medical nutritional therapy (MNT) and structured physical activity interventions play a pivotal role in preventing and managing gestational hypertension and GDM (13, 14). Studies have shown that a balanced diet, including controlled carbohydrate intake and adequate protein consumption, reduces the risk of glucose intolerance and insulin resistance in pregnancy (15, 16). Supervised exercise programs can improve vascular function, glycemic control, and reduce gestational weight gain, mitigating the progression to GDM and hypertensive disorders (17). Furthermore, psychological support and stress management strategies have been associated with improved metabolic profiles and reduced inflammation in high-risk pregnancies (18). Incorporating these interventions into early pregnancy care, guided by AI-driven predictive models, may optimize maternal health and pregnancy outcomes.

This study aims to develop a fusion model integrating ultrasound-based deep learning, radiomics biomarkers, and maternal clinical parameters to predict mid-pregnancy HDP and GDM. By identifying patients at risk for HDP and GDM early in pregnancy, our model could facilitate timely interventions, optimize maternal-fetal health outcomes and guide personalized prenatal care strategies.

## 2 Materials and methods

### 2.1 Patient inclusion

**Figure 1:**
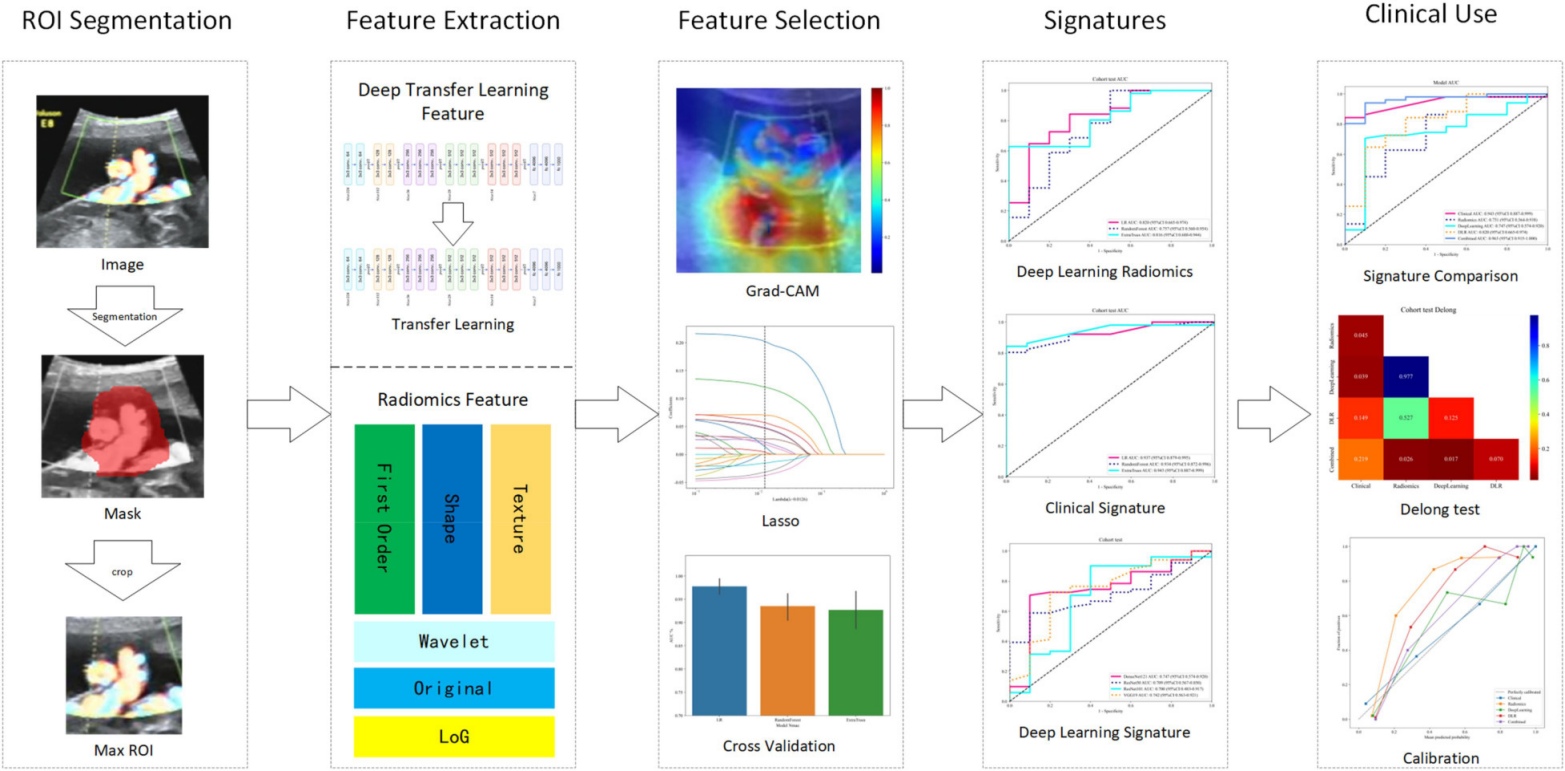
Workflow of this study.

This retrospective diagnostic study included pregnant women who underwent ultrasound screening at 8 weeks of gestation between January 2018 and December 2023. Inclusion criteria were: (1) singleton pregnancy confirmed by ultrasound; (2) gestational age determined by last menstrual period and confirmed by crown-rump length measurement at 8 weeks; (3) complete clinical data; and (4) availability of follow-up data to assess the development of HDP or GDM during the second trimester. Exclusion criteria were: (1) multiple gestation; (2) history of chronic hypertension, pregestational diabetes, renal disease, or autoimmune disorders; (3) missing clinical or ultrasound data; (4) major fetal structural anomalies; and (5) loss to follow-up before 28 weeks of gestation. Clinical and demographic data, including age, body mass index (BMI), medical history, and laboratory findings, were retrospectively collected from electronic medical records. The study workflow is shown in Figure 1. Clinical trial number: not applicable.

### 2.2 Ultrasound collection and preprocessing

All ultrasound examinations were performed using a high-resolution transabdominal ultrasound system (Voluson E10, GE Healthcare) equipped with a 3–9 MHz convex probe. The standard scanning protocol included acquisition of grayscale B-mode images of the gestational sac, yolk sac, and embryo in sagittal and transverse planes. Biometric parameters, including crown-rump length, gestational sac diameter, and yolk sac diameter, were recorded. Doppler imaging was used to assess early placental perfusion and maternal-fetal circulation, including uterine artery pulsatility index. Image preprocessing involved standardization of grayscale intensity, resolution normalization to 256 × 256 pixels, and conversion to grayscale for deep learning and radiomics analysis.

### 2.3 Multi-dimensional health management

For medical nutritional therapy, all participants received standardized dietary counseling at their first-trimester visit. Registered dietitians provided individualized nutritional plans based on maternal weight, body mass index (BMI), and dietary habits. Macronutrient distribution was adjusted according to guidelines for gestational metabolic health, with emphasis on complex carbohydrates, fiber intake, and limited saturated fats. Patients at higher risk of gestational diabetes were encouraged to follow a modified carbohydrate restriction plan with glycemic load control. For physical activity intervention, participants were counseled on maintaining an appropriate level of physical activity during pregnancy. Psychological well-being was assessed using validated stress and anxiety scales, such as the Perceived Stress Scale and the Edinburgh Postnatal Depression Scale, at baseline and during follow-up visits. Participants experiencing moderate to severe stress or anxiety were offered structured psychological support sessions, including cognitive behavioral therapy-based interventions, mindfulness training, and guided relaxation techniques.

### 2.4 Radiomics analysis

Radiomics feature extraction was performed using the Pyradiomics library. Segmented regions of interest (ROI) included the gestational sac and embryo, with features extracted from grayscale intensity, texture, shape, and wavelet-transformed domains. A total of 1,562 radiomics features were initially computed per ROI. Dimensionality reduction was achieved through variance thresholding, correlation analysis, and least absolute shrinkage and selection operator (LASSO) regression to identify the most predictive features.

### 2.5 Deep learning analysis

A convolutional neural network (CNN) was developed to automatically extract deep learning features from the ultrasound images. The network architecture was based on DenseNet121, ResNet50, ResNet101, and VGG19, pre-trained on ImageNet and fine-tuned with the study dataset. The model was trained using a cross-entropy loss function and Adam optimizer with an initial learning rate of 0.001. Data augmentation techniques, including rotation, flipping, and contrast normalization, were applied to enhance generalizability. A feature map visualization technique, such as Grad-CAM, was used to interpret the CNN's focus regions. Deep learning features from the penultimate layer of the trained model were extracted and combined with radiomics and clinical features for further feature selection and model construction.

### 2.6 Model construction

Four predictive models were constructed to evaluate the contribution of different data modalities: (1) Clinical Model: using logistic regression classifier, incorporating maternal age, BMI, blood pressure, glucose levels, and other relevant clinical parameters; (2) Radiomics Model: using a logistic regression classifier trained on selected radiomics features; (3) Deep Learning Model: a CNN-based model trained on ultrasound images for automatic feature extraction and classification; (4) Fusion Model: A multimodal fusion model incorporating all three feature sets. Feature-level fusion was performed by concatenating the radiomics, CNN-extracted, and clinical features before input them into a fully connected layer for final classification.

### 2.7 Statistical analysis

All statistical analyses were performed using R (version 4.1.0) and Python (version 3.8). Baseline characteristics between groups (with and without gestational hypertension or diabetes) were compared using the student's t-test or Mann–Whitney U test for continuous variables and the chi-square or Fisher's exact test for categorical variables. A two-tailed *p*-value < 0.05 was considered statistically significant. Model performance was evaluated using area under the curve, sensitivity and specificity. Calibration curves and decision curve analysis were performed to assess the clinical utility of each model. Deep learning model development and training were accelerated using an NVIDIA GeForce RTX 4090 GPU, while conventional machine learning algorithms were executed on an Intel Core i9-14900K CPU with 128 GB RAM.

### 2.8 Patient characteristics

A total of 213 patients were enrolled, including 47 with adverse outcomes and 166 with non-adverse outcomes (Table 1). Baseline characteristics such as fasting blood glucose (FBG) [median 5 mmol/L in both groups, *p* = 0.457], weight gain [median 12 kg in both groups, *p* = 0.479], age distribution (*p* = 0.259), and body mass index (BMI) categories (*p* = 0.759) showed no significant differences between two groups. However, significant differences emerged regarding hypertension status; 40.4% of patients in the adverse outcome group were hypertensive compared to only 9.6% in the non-adverse group (*p* < 0.001). Lifestyle and intervention adherence varied notably; a significantly lower proportion of patients with adverse outcomes attended weekly lectures (78.7% vs. 97%, *p* < 0.001), participated in psychological counseling (76.6% vs. 98.2%, *p* < 0.001), and completed online tutoring (17% vs. 3%, *p* = 0.001). Moreover, regular exercise (five times per week) was paradoxically more common among patients experiencing adverse outcomes (83%) compared to those without adverse outcomes (4.2%, *p* < 0.001).

**Table 1:** Baseline characteristics of patients between two groups.

Characteristics	Adverse outcome	Non-adverse outcome	*P* value
*n*	47	166	
FBG, median (IQR)	5 (4.6, 5.8)	5 (4.6, 5.675)	0.457
Weight gain, median (IQR)	12 (9, 12)	12 (9, 12)	0.479
Age, *n* (%)			0.259
<30	21 (44.7%)	78 (47.0%)	
≥30; <35	16 (34.0%)	68 (41.0%)	
≥35	10 (21.3%)	20 (12.0%)	
BMI, *n* (%)			0.759
<20	4 (8.5%)	14 (8.4%)	
≥20; <24	20 (42.6%)	84 (50.6%)	
≥24; <28	20 (42.6%)	61 (36.7%)	
≥28	3 (6.3%)	7 (4.3%)	
BP, *n* (%)			<0.001
Normal	28 (59.6%)	150 (90.4%)	
Hypertension	19 (40.4%)	16 (9.6%)	
Exercise 5 times a week, *n* (%)			<0.001
Yes	39 (83.0%)	7 (4.2%)	
No	8 (17.0%)	159 (95.8%)	
Weekly lectures, *n* (%)			<0.001
Yes	37 (78.7%)	161 (97.0%)	
No	10 (21.3%)	5 (3.0%)	
Psychological counseling, *n* (%)			<0.001
Yes	36 (76.6%)	163 (98.2%)	
No	11 (23.4%)	3 (1.8%)	
Completing online tutoring, *n* (%)			0.001
Yes	8 (17.0%)	5 (3.0%)	
No	39 (83.0%)	161 (97.0%)	

### 2.9 Univariable and multivariable analysis

**Figure 2:**
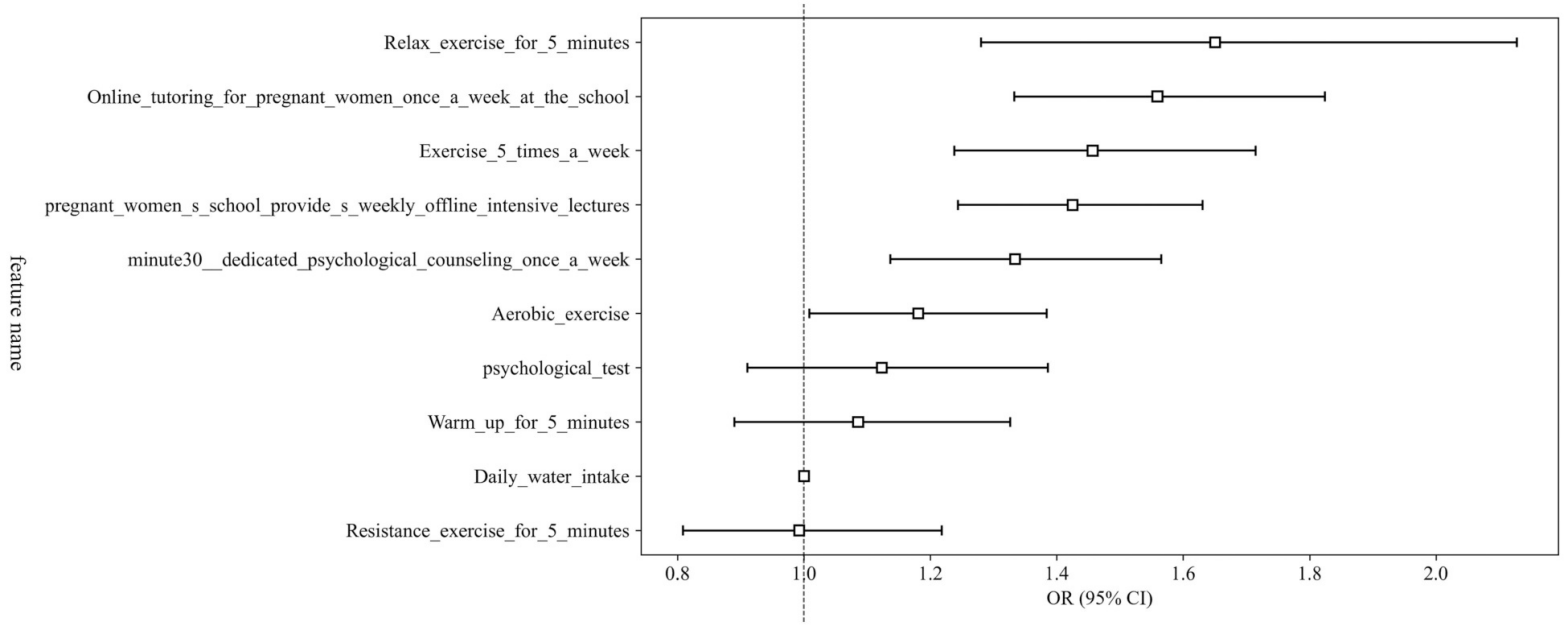
OR of clinical features in multivariable analysis.

We conducted extensive univariable logistic regression analyses to screen clinical variables potentially associated with HDP or GDM. Variables such as psychological testing (OR = 1.507, *p* < 0.05), resistance exercise (OR = 1.783, *p* < 0.05), online tutoring frequency (OR = 1.783, *p* < 0.05), aerobic exercise frequency (OR = 1.929, *p* < 0.05), and relaxation exercises (OR = 2.268, *p* < 0.05) showed statistically significant association with pregnancy complications. Multivariable analysis identified dedicated psychological counseling (OR = 1.333, *p* < 0.05), weekly offline pregnancy classes (OR = 1.425, *p* < 0.05), regular weekly exercises (OR = 1.456, *p* < 0.05), and relaxation exercises (OR = 1.650, *p* < 0.05) as independently significant predictors, indicating their clinical relevance for subsequent model integration (Figure 2).

### 2.10 Deep learning analysis

**Figure 3:**
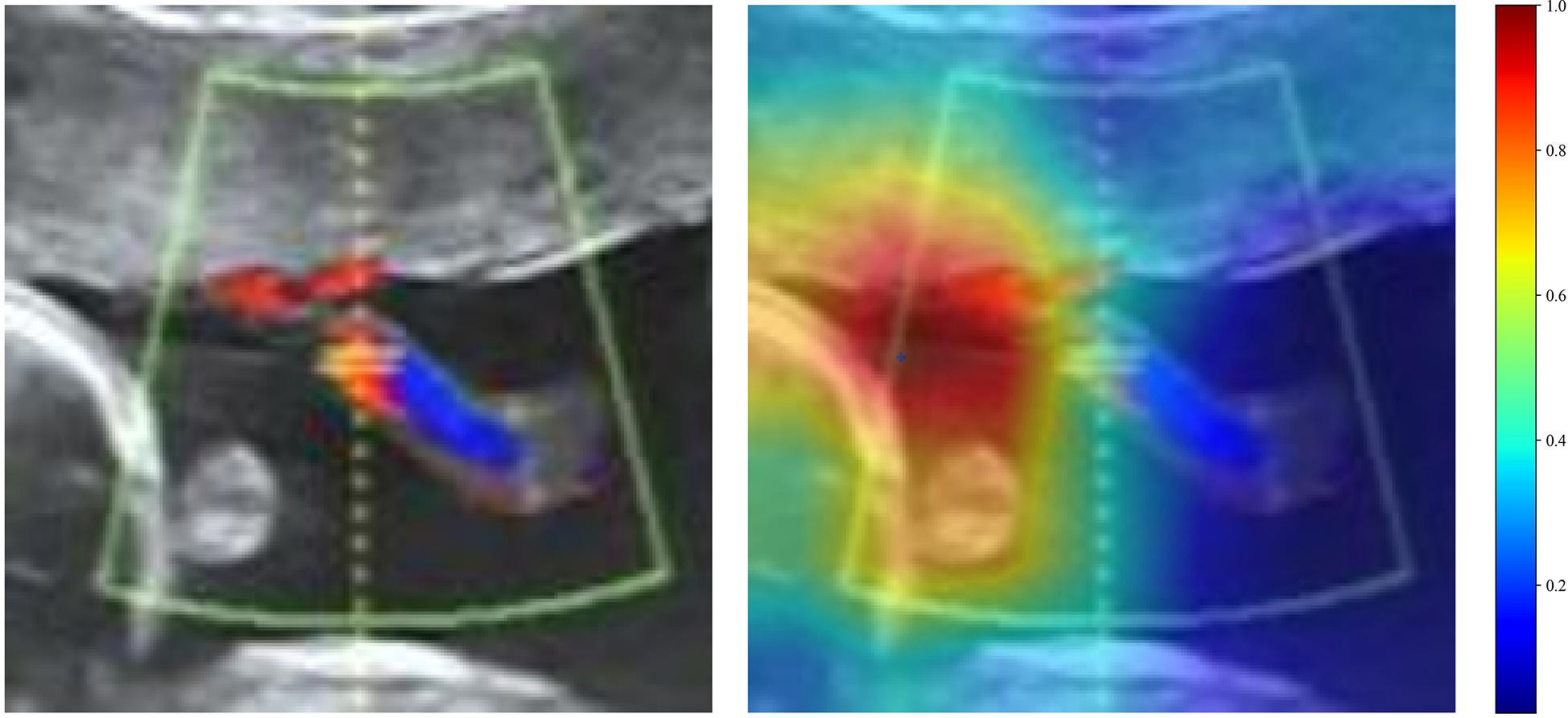
The grad-CAM visualizations for two typical samples, identified as “1” and “26.” these visualizations are instrumental in demonstrating how the model focuses on different regions of the images for making its predictions.

In the deep learning analyses, various CNN architectures including DenseNet121, ResNet50, ResNet101, and VGG19 were tested. DenseNet121 outperformed the other CNN architectures. In the training cohort, DenseNet121 achieved an AUC of 0.924 (95%CI: 0.865–0.983), demonstrating excellent predictive capability. The sensitivity was 100%, specificity was 72.4%, PPV was 93.4%, and NPV was 100%. In the testing cohort, DenseNet121's performance slightly declined but remained robust, with an accuracy of 0.787 and AUC of 0.747 (95%CI: 0.574–0.920), indicating great generalizability. Sensitivity and PPV remained high (90.2% and 85.2%, respectively), although specificity (20%) and NPV (28.6%) were lower. Grad-CAM visualization further elucidated the interpretability of the deep learning model, highlighting specific ultrasound regions integral to predictive outcomes (Figure 3). Regions of high relevance were visually confirmed, providing transparency to the deep learning features and potential clinical interpretability.

### 2.11 Radiomics analysis

**Figure 4:**
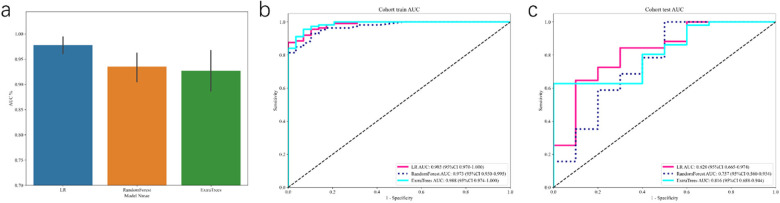
Receiver operating characteristic (ROC) curves for the deep learning signature. The figure presents **(a)** the performance from 5-fold cross-validation, followed by the ROC curves on the **(b)** training and **(c)** independent test cohorts, respectively.

From ultrasound images obtained at 8 weeks of pregnancy, we extracted 1,562 radiomics features, including first-order intensity, shape, and texture features. Feature selection using LASSO regression identified a subset of highly predictive radiomics features. We evaluated several classifiers, including logistic regression (LR), RandomForest, and ExtraTrees, based on ultrasound-derived deep learning and radiomics features. Logistic regression outperformed other models, demonstrating superior predictive accuracy with an AUC of 0.985 (95%CI: 0.970–1.000) in the training cohort, reflecting perfect discriminatory capability. Performance remained robust in the independent testing cohort with an AUC of 0.820 (95%CI: 0.665–0.974), suggesting excellent model generalizability (Figure 4). In contrast, RandomForest and ExtraTrees models showed overfitting, with marked reductions in AUC from training to testing cohorts (RandomForest: training AUC 0.973, testing AUC 0.757; ExtraTrees: training AUC 0.988, testing AUC 0.816).

### 2.12 Fusion model construction and comparasion

To optimize predictive performance, we integrated clinical, radiomics, and deep learning features. Initially, we developed a clinical model incorporating lifestyle factors, exercise routines, psychological interventions, and dietary habits, achieving robust predictive performance (AUC, 0.943; 95% CI: 0.887–0.999) in test cohort. The radiomics model, constructed using selected radiomics features and the ExtraTrees classifier, yielded an AUC of 0.751 (95% CI: 0.564–0.938) in the test set. Concurrently, a deep learning model leveraging DenseNet-121 architecture achieved an AUC of 0.747 (95% CI: 0.574–0.920) in the test set.

**Figure 5:**
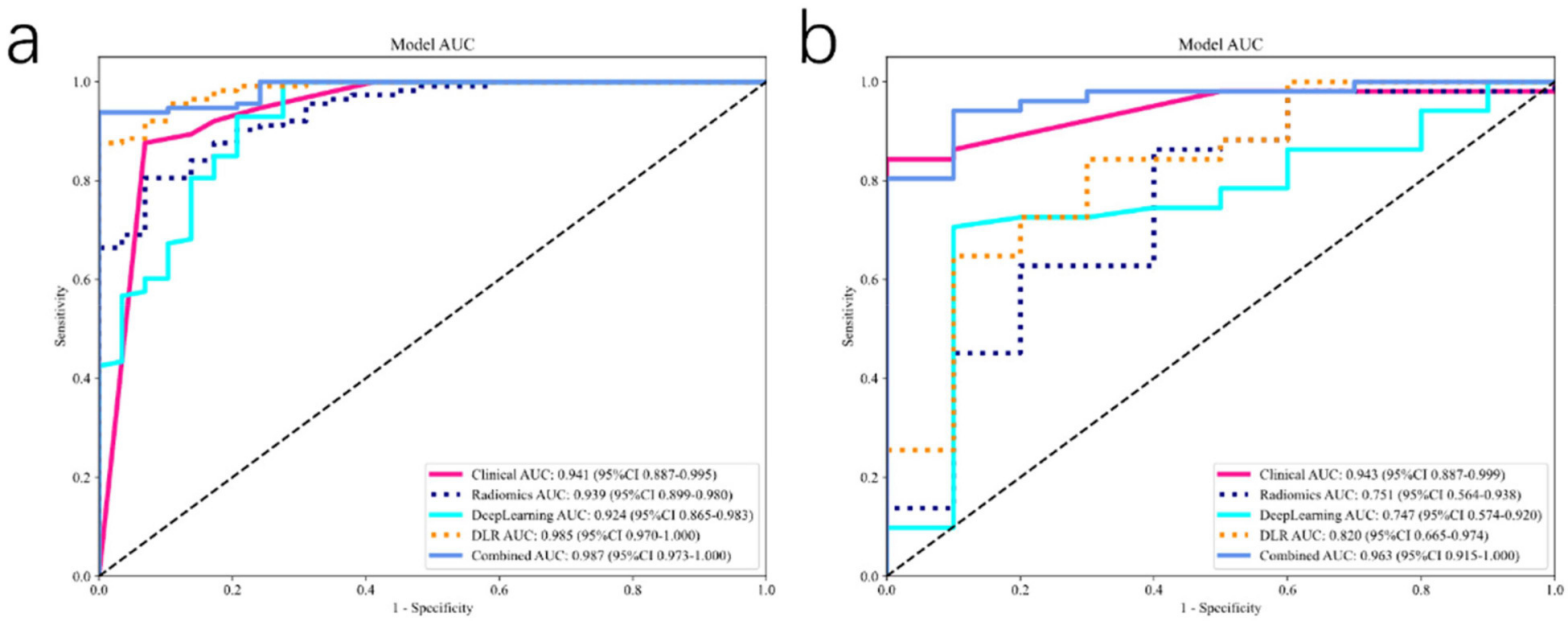
The receiver operating characteristic (ROC) curves for different signatures across train **(a)** and test **(b)** cohorts, offering a visual comparison of their diagnostic abilities.

To further enhance predictive accuracy, we combined these distinct data sources into an integrated Fusion Model, merging clinical characteristics with deep learning and radiomic signatures. The combined model demonstrated significantly improved discriminatory capacity, achieving an AUC of 0.987 (95% CI: 0.973–1.000) in the training cohort and maintaining high performance with an AUC of 0.963 (95% CI: 0.915–1.000) in the test cohort, surpassing individual modalities (Figure 5). Calibration analyses indicated excellent model reliability, with Hosmer-Lemeshow statistics of 0.325 (training cohort) and 0.197 (test cohort), demonstrating close agreement between predicted and observed risk. Statistical comparison using the DeLong test confirmed that the fusion model significantly outperformed the individual radiomics and deep learning models (all *p* < 0.05).

### 2.13 Clinical validation of models

To evaluate the clinical applicability of the predictive models, we performed validation using an independent cohort of pregnant women assessed at 8 weeks of gestation. The performance of the clinical, radiomics, deep learning, and combined models was thoroughly compared. Specifically, the fusion model, integrating clinical characteristics, radiomic signatures, and deep learning-derived imaging features, exhibited superior predictive capability. This represented notable improvement compared with the individual models, affirming the additive value of incorporating diverse sources of data into predictive modeling.

The fusion model demonstrated optimal sensitivity (100%) and acceptable specificity (83.6%) in the test cohort, underscoring its practical utility for clinical screening purposes. High sensitivity is particularly crucial for clinical scenarios where early identification of women at risk for GH or GDM is vital for timely preventive measures and interventions. The high NPV further indicates the combined model's strength in accurately identifying women unlikely to develop these complications, thus helping clinicians effectively allocate medical resources. Decision curve analysis further emphasized the substantial clinical net benefit conferred by the fusion model, affirming its applicability to prenatal healthcare practice.

## 3 Discussion

HDP and GDM are among the most common complications affecting pregnant women, leading to increased maternal and fetal morbidity and mortality. Early prediction and timely intervention remain critical for mitigating adverse pregnancy outcomes. Traditional risk stratification relies on maternal demographic and clinical characteristics, such as age, body mass index (BMI), family history, and metabolic markers, which often fail to capture complex physiological changes occurring early in pregnancy. In this study, we developed a fusion model incorporating first-trimester ultrasonographic deep learning features, radiomic signatures, and clinical parameters to predict mid-pregnancy hypertension or GDM, offering a novel and integrative approach to risk assessment.

Ultrasound imaging during early pregnancy provides a non-invasive and cost-effective modality, widely used for fetal viability and health evaluation (19). However, conventional ultrasound interpretation relies largely on qualitative assessments and is subject to interobserver variability. Compared to ultrasonographer's subjective assessment, deep learning and radiomics tools have demonstrated superior predictive power by leveraging complex spatial and textural information that is imperceptible to the human eye (20). The incorporation of deep learning-based feature extraction enables an objective and automated analysis of high-dimensional image data, enhancing diagnostic consistency and predictive accuracy. Radiomics further complements deep learning approaches by quantifying subtle texture patterns and tissue heterogeneity, providing biomarkers that reflect tissue microstructure, perfusion, and metabolic function related with hypertensive disorders and GDM. The ability of our fusion model to accurately predict the combined outcome of HDP and GDM may be attributed to its capacity to detect these subtle, shared imaging signatures of underlying placental and vascular dysfunction, which aligns with the common pathophysiological basis of these conditions. Several studies have demonstrated the efficacy of deep learning and radiomics in predicting obstetric complications, including preeclampsia and fetal growth restriction, underscoring the potential of image-based biomarkers in maternal-fetal medicine (21, 22). Compared to traditional clinical models, the integration of deep learning and radiomics enhances predictive accuracy and facilitates personalized risk stratification.

Beyond predictive modeling, the clinical impact of early identification extends to targeted intervention strategies, including MNT, structured exercise programs, and psychological support (23). MNT, particularly for high-risk women, plays a pivotal role in optimizing maternal glucose metabolism and blood pressure regulation. Emerging evidence suggests that early dietary modifications, such as a low-glycemic diet and appropriate caloric intake adjustments, can significantly reduce the incidence of GDM (24). Similarly, structured exercise programs tailored to pregnant women have been shown to mitigate the risks of both GDM and hypertensive disorders by improving insulin sensitivity and cardiovascular function (17). Furthermore, psychological counseling and stress management strategies may contribute to improved pregnancy outcomes by modulating maternal stress-related hormonal responses, which are implicated in both hypertensive and metabolic disorders during pregnancy (25). By integrating our model into routine prenatal screening, high-risk women can be identified as early as 8 weeks of gestation, allowing obstetricians to initiate MNT, exercise prescriptions, and psychological support in a timely manner to mitigate complications.

From a clinical perspective, the implementation of AI-driven predictive models holds substantial promise. In comparison to biochemical markers, which often require laboratory testing and may exhibit gestational variability, ultrasound-based tools offer a real-time, non-invasive, and widely accessible solution. Integrating deep learning and radiomics into routine prenatal screening could provide obstetricians with a more refined risk assessment tool, complementing standard laboratory and biometric evaluations. Moreover, our model's ability to identify high-risk pregnancies at an early gestational stage provides an extended window for targeted interventions, potentially improving maternal and neonatal outcomes. This approach aligns with the ongoing shift toward precision medicine, allowing for individualized monitoring and tailored intervention plans. However, several challenges remain before widespread clinical adoption can be achieved. Model generalizability requires validation across diverse populations with varying ethnic and socioeconomic backgrounds. Additionally, the black-box nature of deep learning necessitates efforts to enhance model interpretability, ensuring that predictions align with established pathophysiological mechanisms. Future research should also explore real-time ultrasound image acquisition and automated feature extraction workflows to streamline integration into clinical practice.

Our study has several limitations. First, variability in ultrasound acquisition protocols across institutions may introduce model bias, underscoring the need for standardization in image collection and preprocessing. Secondly, although deep learning models excel in pattern recognition, their black-box nature poses challenges for clinical explainability, necessitating further refinement in interpretable AI tools. Another limitation is the reliance on mid-pregnancy outcomes, which, although clinically relevant, may not fully capture long-term maternal and neonatal sequelae. Future studies should explore the longitudinal impact of early AI-based risk stratification on pregnancy outcomes and postnatal health.

In conclusion, our study demonstrates the feasibility of integrating deep learning, radiomics biomarkers, and clinical factors into a robust predictive model for gestational hypertension and GDM. The model's performance in predicting this combined outcome is supported by the overlapping pathophysiology of these disorders. This approach offers a non-invasive, early risk assessment tool with clinical utility, facilitating personalized prenatal care and timely intervention. Future study should focus on expanding external validation and integrating AI-driven predictions into clinical decision support systems to further improve obstetric care.

## 4 Limitations

This study has several limitations that should be acknowledged. First and foremost, the single-center, retrospective design is a key limitation. While we implemented rigorous internal validation with a randomized train-test split, which showed no significant demographic differences (Table 1), the generalizability of our findings remains to be verified. The patient population, imaging protocols, and clinical practices at a single institution may introduce inherent biases and may not be fully representative of other centers or broader demographic groups. Therefore, external validation on multi-center and prospective cohorts is essential to confirm the robustness and clinical applicability of our proposed model before it can be widely adopted.

## Data Availability

The raw data supporting the conclusions of this article will be made available by the authors, without undue reservation.
